# A retrospective observational study on the efficacy of colistin by inhalation as compared to parenteral administration for the treatment of nosocomial pneumonia associated with multidrug-resistant *Pseudomonas aeruginosa*

**DOI:** 10.1186/1471-2334-11-317

**Published:** 2011-11-15

**Authors:** Reinout Naesens, Erika Vlieghe, Walter Verbrugghe, Philippe Jorens, Margareta Ieven

**Affiliations:** 1 Laboratory of Medical Microbiology, Antwerp University Hospital, Edegem, Belgium; 2 Department of Tropical Diseases, Antwerp University Hospital, Edegem, Belgium; 3 Intensive care unit, Antwerp University Hospital, Edegem, Belgium

## Abstract

**Background:**

Colistin is used as last treatment option for pneumonia associated with multidrug-resistant (MDR) *Pseudomonas *spp.. Literature about the best administration mode (inhalation versus parenteral treatment) is lacking.

**Methods:**

A retrospective study of 20 intensive care patients with a pneumonia associated with MDR *P. aeruginosa *receiving colistin sulphomethate sodium (Colistineb^®^) between 2007 and 2009 was performed. A strain was considered multidrug-resistant if it was resistant to at least 6 of the following antibiotics: piperacillin-tazobactam, ceftazidime, cefepime, meropenem, aztreonam, ciprofloxacin, and amikacin. The administration mode, predicted mortality based on the SAPS3 score, SOFA score at onset of the colistin treatment, clinical and microbiological response, and mortality during the episode of the infection were analysed. The non parametric Kruskal-Wallis and Fisher's Exact test were used for statistical analysis of respectively the predicted mortality/SOFA score and mortality rate.

**Results:**

Six patients received colistin by inhalation only, 5 were treated only parenterally, and 9 by a combination of both administration modes. All patients received concomitant beta-lactam therapy. The mean predicted mortalities were respectively 72%, 68%, and 69% (p = 0.91). SOFA scores at the onset of the treatment were also comparable (p = 0.87). Clinical response was favorable in all patients receiving colistin by inhalation (6/6) and in 40% (2/5) of the patients receiving colistin parenterally (p = 0.06). In the patients with colistin administered both via inhalation and parenterally, clinical response was favorable in 78% of the patients (7/9) (p = 0.27 as compared to the treatment group receiving colistin only parenterally). When all patients with inhalation therapy were compared to the group without inhalation therapy, a favorable clinical response was present in respectively 87% and 40% (p = 0.06). In none of the patients, the *Pseudomonas *spp. was eradicated from the follow-up cultures.

All patients in the parenterally treated group died. None of the patients receiving colistin by inhalation, and 3 of 9 patients of the combination group eventually died (p = 0.002 and p = 0.03 respectively as compared to the group receiving colistin only parenterally).

**Conclusions:**

Aerosolized colistin could be beneficial as adjunctive treatment for the management of pneumonia due to MDR *P. aeruginosa*.

## Background

According to data from the US Centres for Disease control and Prevention (CDC) and the National Nosocomial Infection Surveillance System (NNISS), *Pseudomonas aeruginosa *is the second most common cause (17%) of nosocomial pneumonia, and more importantly the most common multidrug-resistant (MDR) gram-negative pathogen causing pneumonia in hospitalized patients [1]. The increased prevalence of MDR and panresistant *P. aeruginosa *and *Acinetobacter *spp. strains in multiple parts of the world has created a new antibiotic therapeutic void, leading to the use of the neglected class of polymyxins [1]. Until recently, the polymyxin class was mainly used via inhalation to treat high-density respiratory tract colonization due to MDR *P. aeruginosa *in patients with cystic fibrosis since this class was thought to be unacceptably toxic when administered parenterally. However, in recent years, colistin was observed to be probably less toxic than previously proposed, and to offer an acceptable efficacy for treatment of severe infections due to MDR gram-negative bacteria [2]. Colistin is now being used increasingly as a last treatment option for treatment of nosocomial pneumonia with MDR gram-negative bacteria [2].

We report our 2-year experience with the use of colistin for the treatment of 20 patients, admitted to our intensive care unit (ICU), who developed nosocomial pneumonia associated with MDR *P. aeruginosa*.

## Methods

This retrospective study was performed at the Antwerp University Hospital, the 573 bed tertiary referral hospital of the University of Antwerp. Pharmacy records were reviewed to identify patients who were admitted to the 39 bed ICU, and were receiving colistin (Colistineb^®^, colistin sulphomethate sodium, Forest Laboratories, Kent, UK) between June 2007 and June 2009 for therapy of nosocomial pneumonia associated with MDR *P. aeruginosa*. The primary objective was to compare the treatment outcome of the pneumonia (clinical success; mortality) between different colistin treatment groups (parenteral treatment, inhalation treatment, combination of parenteral and inhalation treatment). The study was approved by the ethical committee (ref. 10/22/154) of the hospital which judged that an informed consent was not required. The following data were recorded for each patient: age, sex, predicted mortality rate based on the Simplified Acute Physiology 3 Score (SAPS3) - score, Sequential Organ Failure Assessment (SOFA) - score at admission to the ICU, and at the onset of the colistin therapy, concomitant other infections, length and dosage of colistin therapy, length of stay, time between admission and development of the pneumonia, simultaneous use of other antibiotics, clinical and microbiological response, and mortality during the episode of the infection [3,4]. An automated Patient Data Management system (PDMS from iMD-soft) was used to collect most of the data.

Diagnosis of nosocomial pneumonia was defined as pneumonia that occured ≥ 48 hours after hospital admission. Pneumonia was diagnosed by the finding of a new pulmonary infiltrate associated with at least two of the following criteria: fever > 38°C, leukocytosis > 11,000 cells/mm^3^, or purulent respiratory secretions [5]. Ventilator associated pneumonia (VAP) was defined as a nosocomial pneumonia in a patient with at least 48 hours of mechanical ventilatory support [5]. The etiology of the pneumonia was established by isolation of the organism from blood cultures, or by culturing the organism from endotracheal aspirates or mini-BALs irrespective of the bacterial count.

The antibiotic regimen was determined by the primary treating ICU physician on the basis of a standardised dose regimen, the clinical follow up and response. Generally, ICU physicians decided to treat patients arbitrarily with parenterally administered colistin, together or without inhalation, when they estimated that the infection was "more severe" or if another infection focus with *P. aeruginosa *was present. Patients were excluded for analysis if they received ≤ 2 days of colistin therapy. A favorable clinical response was defined as a resolution of presenting symptoms and signs at the end of the treatment, and an unfavorable clinical response was defined as persistence or worsening of presenting symptoms and signs [6]. Favorable microbial response was defined as eradication of the strain in a respiratory sample collected in the period of 1 day before until 7 days after discontinuation of the colistin therapy.

Renal function was monitored by serial measurement of the serum creatinine level. Creatinine at the onset of the colistin therapy, maximal creatinine during the colistin therapy, and prior renal replacement therapy (RRT) were recorded. Renal failure was defined following the RIFLE criteria as described by Hartzell et al [7]. Neurotoxicity rates were not determined because of the lack of objective criteria in our patients.

Bacterial isolates were identified by biochemical manual testing methods. Susceptibility testing, including susceptibility for colistin, was performed using disk diffusion testing (Sensitabs, Rosco diagnostics, Denmark) using Clinical Laboratory and Standards Institute (CLSI) breakpoints [8]. Multidrug-resistance was defined as non-susceptibility to at least six of the following antibiotics: meropenem, amikacin, piperacillin-tazobactam, ceftazidime, cefepime, aztreonam, and ciprofloxacin [9]. Quality control was performed weekly in the study period since 2007 using ATCC 27853, and was always situated between reference values.

Colistin was administered intravenously at a dosage of 62500 IU/kg/day in 3 to 4 divided doses, and adjusted for creatinine clearance if necessary. If colistin was administered by inhalation, a standardised regimen of 2 mIU/aerosol t.i.d. was administered. In mechanically ventilated patients, the efficient administration of the inhaled/aerosolized colistin was guaranteed by the use of the standard nebulizers for the ventilators used: Servo Ultra Nebulizer 145/345 for the Servo-I (Maquet, Solna, Sweden) and the 84 12 935 nebulizer for the Dräger Evita 4 Ventilator (Dräger, Lennik, Belgium). During spontaneous breathing, optimal aerosol particle deposition was executed by standard delivery system (Cirrus drug nebulizer, Intersurgical, Berkshire, UK).

All data were entered into a database and analyzed using SAS 9.2. (SAS Institute Inc., North Carolina, USA). For the continuous data, the non-parametric Kruskall Wallis test was used. For the categorical data, we used the non-parametric Fisher's Exact test.

## Results

Between June 2007 and June 2009, 20 patients who developed nosocomial pneumonia with MDR *P. aeruginosa *were treated with colistin (> 2 days) at our ICU: 9 patients received a combination therapy of both parenteral and inhalation therapy, 6 were treated by inhalation only, and 5 only parenterally (Table 1). MDR *P. aeruginosa *was recovered from the respiratory tract in all patients and additionally from the blood cultures in 2 patients. 4 patients were excluded from analysis since they received colistin for ≤ 2 days. Ten of 20 strains were panresistant, except for their susceptibility to colistin whereas 6 of 20 (30%) strains were susceptible to piperacillin-tazobactam, 3 (15%) to cefazidime, and 1 (5%) to amikacin. All patients received combination therapy with beta-lactams, of which piperacillin-tazobactam (n = 12), and meropenem (n = 9) were most frequently used. Piperacillin-tazobactam was switched to meropenem in one patient during colistin therapy. Six out of 20 (30%) of the nosocomial pneumonias were considered VAPs according to the definition [5]. Five out of 9 (56%) patients on combination therapy also suffered from an infection associated with *P. aeruginosa *at another focus (one intra-abdominal infection, and four skin and soft tissue infections). In one of the 5 patients with parenteral colistin therapy, an intra-abdominal infection with *P. aeruginosa *was present. One patient in the inhalation group, developed an *Enterobacter *sepsis, which was treated accordingly. The age, gender distribution, and prior clinical condition were comparable between the three different groups (Table 1). The mean predicted mortalities based on the SAPS3 score were respectively 72%, 68%, and 69% (p = 0.91) [4]. SOFA scores at admission and at the onset of the treatment were also comparable (p = 0.18 and p = 0.87 respectively) [4]. Mean treatment durations were comparable and ranged from 19 to 27 days (p = 0.87, Table 1). In all but one patient, follow up cultures were performed. In none of them, microbiological eradication was achieved (Table 2).

**Table 1:** Patient characteristics of the different treatment groups

	Inhalation	Parenteral + Inhalation	Parenteral	All patients treated by Inhalation	p-value (difference between groups?)
Number of patients (medical-surgical)	6 (1-5)	9 (5-4)	5 (3-2)	15	/

Age (years)	62.5 (15-84)	67.9 (59-76)	64.8 (46-77)	65.7 (15-84)	0.91

Gender (Male %)	66.7%	77.8%	60.0%	58%	0.83

SOFA-score at admission	5.2 (2-9)	6.4 (0-15)	10.0 (3-13)	5.9 (2-15)	0.18

SOFA-score start colistin	6.3 (1-15)	6.7 (2-11)	6.0 (3-9)	6.5 (1-15)	0.87

SAPS3-score	80.7 (70-88)	80.8 (70-95)	79.0 (73-85)	80.7 (70-95)	0.92

Length of stay (days)	55.0 (19-103)	73.0 (16-141)	40.0 (29-64)	65.8 (16-141)	0.16

Time between admission and development of the pneumonia (days)	28.0 (7-75)	25.1 (8-68)	19.6 (13-29)	16.7 (7-75)	0.99

Treatment duration (days)	27.2 (6-96)	19.3 (3-46)	21.0 (9-28)	22.5 (3-96)	0.87

Creatinine at onset colistin; RRT patients excluded (mg/dL)	0.8 (0.7-1.2)	2.3 (0.9-4.7)	0.9 (0.8-1.0)	1.6 (0.7-4.7)	0.39

Creatinine during colistin therapy; RRT patients exluded (mg/dL)	1.0(0.6-1.3)	2.6 (1.1-5.8)	1.9 (1.9-2.0)	1.9 (0.6-5.8)	0.36

Number of RRT patients	2/6	4/9	2/5	6/15	/

Data are presented as mean values with the range between brackets. RRT: renal replacement therapy.

**Table 2:** Outcome of the different treatment groups

Treatment groups	Number of patients with *P. aeruginosa *susceptible to colistin AND one beta-lactam	Clinical failure when only susceptible to colistin	Clinical failure when susceptible to colistin and at least one other antibiotic agent	Microbiological failure	Favorable clinical response	Mortality
Inhalation	4/6	0/2	0/4	5/5 1 no control data	6/6	0/6

Inhalation + Parenteral	3/9; 1/9 was susceptible to aminoglycosides	1/5	1/4	9/9	7/9	3/9

Inhalation and inhalation + parenteral	8/15	1/7	1/8	14/14	13/15	3/15

Parenteral	2/5	2/3	1/2	5/5	2/5 p = 0.06 as compared to the inhalation group p = 0.27 as compared to the parenteral + inhalation group p = 0.07 as compared to both the inhalation and inhalation + parenteral group	5/5 p = 0.002 as compared to the inhalation group; p = 0.03 as compared to the parenteral + inhalation group; p = 0.003 as compared to both the inhalation and inhalation + parenteral group

Clinical response was favorable in all patients treated by inhalation only (6/6) and in 40% (2/5) of the patient treated only parenterally (p = 0.06). In the patients with the combination colistin therapy, clinical response was favorable in 78% (7/9) (p = 0.27 as compared to the treatment group with only parenterally administered colistin). When all patients with inhalation therapy (inhalation only or concomitant parenteral treatment) are compared to the group without inhalation therapy, favorable clinical response was present in respectively 87% and 40% (p = 0.06). Antibiotic therapy with colistin clinically failed in 2/10 versus 3/10 of patients with pneumonia associated with MDR *P. aeruginosa *strains susceptible to respectively only colistin versus colistine and at least one other antimicrobiological agent [Table 2]. All patients (5/5) died in the group only receiving colistin parenterally. The mortality was attributed directly to the pneumonia in 3 of 5 patients. The two other patients died during therapy because of 1/an abdominal sepsis of unknown etiology, and 2/concomitant severe pathology. In contrast, none of the patients receiving colistin by inhalation only, and 3 of 9 (one case fatality was directly linked to a cerebrovascular accident) of the patients treated by the combination therapy, eventually died (p = 0.002 and p = 0.03 respectively as compared to the group with colistin administered parenterally only).

The mean duration of treatment was prolonged (> 14 days) in all three groups.

Approximately half of all patients in the different groups received prior RRT (Table 1). Renal toxicity was absent in all the patients (6/6) who did not receive prior RRT, and only received colistin via inhalation. In the group of patients with parenteral monotherapy who did not get prior RRT, all patients (3/3) suffered from "Injury" according to the RIFLE criteria [7] (Table 1). In the group with the combination therapy, 1 of 5 suffered from "Failure" because of a creatinine level of > 4 mg/dL. The failure in this patient was however already present at the onset of the colistin treatment.

Although neurotoxicity rates were not studied in our analysis, no major insults on behalf of the colistin therapy were described in the follow up notes of the different patients. Some cases of critical illness neuropathy were however noticed, but it was unclear whether there was a relationship with the colistin treatment (data not shown).

## Discussion

The clinical response rate of 40% (2/5) in the group with colistin administered parenterally only is in line with results from similar studies in which response rates of 17-73% were reported with the use of colistin in the treatment of nosocomial pneumonia [1,10,11]. Response rates with colistin therapy for treatment of nosocomial pneumonia seem to be lower compared to treatment of nonpulmonary infections, but are comparable to previously reported response rates of nosocomial pneumonia treated with piperacillin, imipenem-cilastatin, and ciprofloxacin [1]. The higher response rate of 100% (6/6) (p = 0.06) in our group treated by inhalation of colistin was probably related to several factors. First: even though the clinical conditions (according to the SOFA and SAPS3 score) of all patient groups were comparable, clinicians in our hospital more easily treated patients parenterally when they estimated that the infection was "more severe" or if another infection focus with *P. aeruginosa *was present. Unfortunately, no validated score is available for the severity of nosocomial pneumonia itself. Secondly, 4 out of 6 strains were susceptible to a beta-lactam which was also part of the patients' antibiotic regimen. The efficacy of this combination therapy however has not yet been proven. Third, the efficacy of aerosolized colistin in pneumonia might have contributed to the result. Pharmacokinetic studies have shown that a single inhalation of 2 million IU of colistin leads to high sputum concentrations of the drug even 12 h after the administration [12]. Guidelines by the American Thoracic Society and the Infectious Diseases Society of America mention that "aerosolized antibiotics may be considered as adjunctive therapy in patients with MDR gram-negatives who are not responding to systemic therapy" [13,14]. Although only very few clinical studies are performed on aerosolized colistin, results look promising: a case series of Falagas et al. showed that 4/5 of the patients who developed a nosocomial pneumonia (*P. aeruginosa *in 2 cases), and who were treated with colistin aerosol in monotherapy, had a favorable clinical evolution [15]. A second study showed a cure rate of even 88% (7/8) in patients with a nosocomial pneumonia treated with a combination therapy of parenteral colistin, and colistin via inhalation (*P. aeruginosa *in 1/8 cases). This was higher, although not statistically significant, than the 67% (30/45) cure rate in patients treated by the parenteral route only [16]. Another small study of only 3 patients showed improvement after addition of aerosolized colistin to the antipseudomonal beta-lactam therapy [17]. In a fourth study, the role of inhalation therapy was suggested as effective salvage therapy for treatment of nosocomial pneumonia caused by MDR gramnegative bacteria (*P. aeruginosa *in 16 of 19 patients) since therapy with aerosolized polymyxin B led to improvement or cure in 13 of 14 (93%) patients in whom previous parenteral therapy with polymyxin B failed [18]. In a retrospective cohort study, Korbila et al. found a cure rate of 80% (62/78) in patients who received a combination of aerosolized and parenteral colistin versus 61% (26/43) in patients who received parenteral colistin alone (p 0.025) [19]. In a prospective study from Michalopoulos et al, a clinical response of VAP was observed in 50/60 (83.3%) patients treated by inhalation therapy. In this study, fifty-seven of the patients received concomitant intravenous treatment with colistin or other antimicrobial agents [20] (Table 3). In our study, 87% (13/15) of the patients treated with a beta-lactam and colistin by inhalation with or without concomitant parenteral treatment, had a favorable outcome, which is higher than the 40% (2/5) when patients only received a beta-lactam and parenteral colistin. Numbers were too low to show significance (p = 0.07). However, significance was reached in the mortality rates (3/15 versus 5/5 respectively; p = 0.003), even though the predicted mortality according to the SAPS-3 score, and the clinical condition according to the SOFA-score at admission and at the onset of the colistin treatment, were comparable. In contrast to the clinical response, no microbiological eradication was noticed in any of the patients. Low ratios of failing microbiological eradication were found by Montero et al. (eradication in only 7 of 13 treated patients for pneumonia). They found that colonization with MDR *Pseudomonas *prior to infection was an indepent risk factor for persistent colonization [21].

**Table 3:** Summary of available studies on colistin administered by inhalation versus administered parenterally

Study name	Route of administration	% of success
Falagas [15]	inhalation	80% (4 of 5)

Michalopoulos [16]	inhalation + parenteral parenteral	88% (7 of 8) 67% (30 of 45)

Hamer [17]	inhalation + parenteral beta-lactam therapy	100% (3 of 3)

Pereira [18]	inhalation after failing parenteral therapy	93% (13 of 14)

Korbila [19]	inhalation + parenteral parenteral	80% (62 of 78) 61% (26 of 43)

Michalopoulos [20]	inhalation + parenteral (57 of 60 patients) inhalation (3 patients)	83% (50 of 60)

The prolonged colistin treatment was mainly due to the fact that colistin was already the last treatment option, and the clinical response was unfavourable in 5 of 20 patients. In addition, the absence of serious adverse events and the persistent colonization of the respiratory tract may have played a role.

Recent studies reported nephrotoxicity rates of 8% to 36% [2]. In our case series, 100% (3/3) of patients treated by parenterally administered colistin, who were not receiving prior RRT, developed nephrotoxicity to be classified as "Injury" according to the RIFLE criteria [7] (Table 1). Of those 3 patients, mean serum creatinine at the onset of the colistin therapy raised from 0.9 mg/dL to a maximum of 1.9 mg/dL during therapy. Since all 3 patients died, follow up of the creatinine levels was not possible in this subgroup. We hypothesise that the high rate of renal injury in this group was not attributable to colistin only, but may also have resulted from renal damage in the setting of a failing therapy, eventually leading to mortality in all patients. In the group with the combination therapy, in which clinical response was favorable in 78% of the patients (7/9), renal "Failure" was only present in one patient according to the RIFLE criteria. The failure in the patient was however already present before the colistin was administered. Similarly, Falagas et al. found a median rise in creatinine levels of only 0.25 mg/dL during prolonged (mean: 43 days) parenteral colistin treatment in 19 courses. Moreover, the levels returned close to baseline at the end of the treatment (0.1 mg/dL). In the group with inhalation therapy, none of the patients developed nephrotoxicity, which was probably attributed to the more "topical" distribution of colistin when delivered by inhalation [22].

Shortcomings and possible confounders in our study were 1/the use of a single centre retrospective analysis on small numbers; 2/the difficulty to discriminate between patients that were infected versus colonized by *Pseudomonas *spp.; 3/the inability of including time to appropriate therapy in the different treatment groups; 4/the administration of concomitant antimicrobial therapy.

## Conclusion

We conclude that aerosolized colistin appears to be a promising adjunct when used in combination with parenteral therapy (colistin or beta-lactams) over parenteral therapy alone. Since colistin is a last-line drug for infections with highly resistant *Pseudomonas *spp., there is an urgent need for randomized, controlled trials to further delineate its role in the ICU setting.

## Abbreviations

CDC: Centers for Disease Control; CLSI: Clinical Laboratory Standards Institute: ICU: Intensive Care Unit; MDR: MultiDrug-Resistant; NNIS: National Nosocomial Infection Surveillance System; PDMS: Patient Data Management System; RRT: Renal Replacement Therapy; SAPS 3 Simplified Acute Physiology 3; SOFA: Sequential Organ Failure Assessment; VAP: Ventilator Associated Pneumonia

## Competing interests

The authors declare that they have no competing interests.

## Authors' contributions

RN carried out the study. All authors participated in the design of the study. WV, PGJ

were also treating clinicians. All authors read and approved the final manuscript.

## Pre-publication history

The pre-publication history for this paper can be accessed here:

http://www.biomedcentral.com/1471-2334/11/317/prepub
